# Examining the Role of Acculturation in E-Cigarette Use among U.S. Immigrant Adults

**DOI:** 10.3390/ijerph18073658

**Published:** 2021-04-01

**Authors:** Sunday Azagba, Lingpeng Shan

**Affiliations:** Department of Family and Preventive Medicine, University of Utah School of Medicine, Salt Lake City, UT 84108, USA; lingpeng.shan@utah.edu

**Keywords:** e-cigarette use, acculturation, immigrants, native-born, length of residence

## Abstract

Evidence suggests that as immigrants’ length of residence in the host country increases, they may integrate their behavior and norms to align with the new community’s cultural norms. The current study examined e-cigarette use among immigrants in the U.S., and whether the length of residence in the U.S. is associated with e-cigarette use among immigrants compared to the native-born population. Data were drawn from the 2014/15 and 2018/19 Tobacco Use Supplement to the Current Population Survey. Multivariable logistic regression was used to compare differences in e-cigarette use between native-born populations and immigrants, when immigrants’ length of residence in the U.S. was considered. Among immigrants, the prevalence of ever and current e-cigarette use increased significantly from 2.5% and 0.5% in 2014/2015 to 3.2% and 0.8% in 2018/2019, respectively. Multivariable analysis showed that immigrants had significantly lower odds of ever e-cigarette use compared to the mainland-born citizen (0–5 years in the U.S., adjusted Odds Ratio (aOR) 0.57, 95% Confidence Interval (CI) 0.46–0.69; 6–10 years, aOR 0.51, 95% CI 0.41–0.63; 11–20 years, aOR 0.45, 95% CI 0.39–0.53; 20+years, aOR 0.68, 95% CI 0.62–0.76). Similar results were found for current e-cigarette use, with immigrants being less likely to be current users. Findings that e-cigarette use among all immigrants—regardless of years living in the U.S.—was consistently lower than among the native-born population run contrary to the notion that as length of stay increases, health behaviors between immigrants and native populations of the host country become similar.

## 1. Introduction

Globally, there has been a changing tobacco product landscape with the introduction of vaping products, such as electronic nicotine vaping systems (commonly referred to as e-cigarettes). E-cigarettes are battery-powered devices that provide nicotine and other additives to the user in the form of an aerosol [1]. Since entering the U.S. market in the middle 2000s, the sales of e-cigarettes in the United States have risen substantially. The percentage of U.S. adults who have ever used an e-cigarette has increased from 3.3% in 2010 to 14.9% in 2018 [2,3]. In 2018, an estimated 7.98 million U.S. adults (3.2%) were current e-cigarette users [2]. As one of the newer tobacco products, the long-term health consequences of e-cigarettes remains unknown. Some studies have shown that e-cigarettes produce fewer harmful chemical components than conventional cigarettes [4,5,6,7]. Although the motivation to quit smoking has partially contributed to the popularity of e-cigarettes among adults [8], evidence supporting the effectiveness of the e-cigarette as a smoking cessation aid is limited [9,10].

A growing number of studies point to e-cigarette use disparities among specific subpopulations, with prior research showing a higher prevalence of e-cigarette use among younger adults, men, and non-Hispanic white adults [2]. There is scant e-cigarette literature among immigrant populations [11,12]. Evidence suggests that as immigrants’ length of residence in the host country increases, they may integrate their behavior and norms to align with the cultural norms of the new community [13,14]. The process is often referred to as acculturation, which has been shown to be positively correlated with the length of residence [13,15,16]. It remains unclear whether e-cigarette use follows similar temporal patterns as those between immigrant or nativity status and smoking [17,18,19,20]. A prior study used the 2014 National Health Interview Survey and found that naturalized citizens and non-citizens had lower odds of ever e-cigarette use compared to U.S. natives [11]. In terms of current e-cigarette use, Wang and colleagues found no significant differences between naturalized U.S. citizens and U.S. natives; however, non-U.S. citizens had lower odds of current e-cigarette use than U.S. natives [11]. Absent from the limited literature are studies comparing e-cigarette use between immigrants’ length of stay and the native-born population. 

The current study examined e-cigarette use among immigrants in the U.S., and whether the length of residence in the U.S is associated with e-cigarette use among immigrants compared to the native-born population, using data from the 2014/2015 and 2018/2019 Tobacco Use Supplement to the Current Population Survey. Several reviews have documented that the role of acculturation in smoking behavior appears more gender-specific [17,21,22]. In a systematic review study, Bethel and Schenker found a consistent increase in smoking prevalence with acculturation measurements among Hispanic women, while mixed results were observed among Hispanic men [17]. Thus, we assessed whether such association holds in both males and females. 

## 2. Materials and Methods

### 2.1. Data

The Tobacco Use Supplement to the Current Population Survey (TUS-CPS) is a large household survey among the civilian, non-institutionalized population who are 16 years of age and older in the United States. It is administered by the Census Bureau and sponsored by the National Cancer Institute (NCI). The CPS is a monthly labor force interview survey conducted in more than 50,000 households across the country. Since 1992, the TUS-CPS has been conducted every three to four years to collect data on tobacco-related products [23]. Since the 2014–2015 survey cycle, the TUS-CPS has expanded to cover e-cigarette use behaviors. The present study sample was determined from the question of citizenship status. The possible answers included: “Native, Born in the United States”, “Native, Born in Puerto Rico or U.S. Outlying Area”, “Native, Born Abroad of American Parent Or Parents”, “Foreign Born, U.S. Citizen by Naturalization”, and “Foreign Born, Not a Citizen of the United States”. In order to limit our analysis to the subpopulation of interest, we included only those who responded as being “Foreign Born, U.S. Citizen by Naturalization” or “Foreign Born, Not a Citizen of the United States” to the citizenship status question. Those who responded “Native, Born in the United States” to the citizenship question were also included in our study to compare e-cigarette use between mainland-born citizens and immigrants. The final analysis included 35,642 immigrants and 261,640 mainland-born citizens who met the inclusion criteria from survey cycles 2014/2015 and 2018/2019.

### 2.2. Measures

The main outcome variables were two dichotomized variables: (1) e-cigarette ever use, and (2) current (some days or every day) e-cigarette use during the survey period. In terms of independent variables, we also obtained age (18–24, 25–34, 35–44, 45–54, or at least 55 years old), sex (male or female), race (non-Hispanic White, non-Hispanic Black, Hispanic, or non-Hispanic other), employment status (full-time, part-time, unemployed, or not in labor force), education attainment (some high school or less, high school graduate or GED (General Educational Development), some college (no degree) or an associate’s degree, or at least a bachelor’s degree), income (USD <25,000, USD 25,000–50,000, or USD >50,000). Variables indicating other tobacco product use and immigrants’ year of arrival in the U.S. were also included in analyses. Number of years in the U.S. was categorized as ≤5, 6–10, 11–20, and 20+. Other tobacco product use was defined as a respondent who had used 100 cigarettes in his/her lifetime and smoked cigarettes some days or every day, or had ever used other tobacco (i.e., filtered cigars, cigarillos, traditional cigars, hookah or water pipe, pipes, or smokeless tobacco, such as moist snuff, dip, spit, chew tobacco, or snus).

### 2.3. Statistical Analysis

The weighted sample demographic characteristics were analyzed by e-cigarette ever use status. Rao–Scott chi-square tests were used to compare characteristics between the two groups. We generated estimates of the prevalence of e-cigarette ever and current use for each year of data for the full sample, as well as by sociodemographic characteristics (age subgroups, sex, race, employment status, income level, education attainment, other tobacco product use, and years in the U.S.). Chi-square tests were used to examine the significance of percentage change in e-cigarette use by sociodemographic characteristics (age subgroups, sex, race, employment status, income level, education attainment, other tobacco product use, and years in the U.S.). Additionally, multivariable logistic regressions were performed to examine the association between immigrants’ length of stay and e-cigarette use compared to mainland-born citizens. We also examined potential gender differences by stratifying analyses by sex. Analyses were adjusted for covariates, including age, sex, race, employment status, income level, education attainment, and other tobacco product use. Sampling weights were included in all analyses to account for the complex survey design. All tests were two-sided, and a *p*-value of <0.05 was considered significant. We performed all data analyses using Statistical Analysis System (SAS) version 9.4 (SAS Institute, Inc., Cary, NC, USA).

## 3. Results

The sample characteristics of immigrants included in the study are shown in Table 1. Of the 35,642 immigrants included in the analyses, 29.1% were at least 55 years old, 49.2% were male, and 45.8% were Hispanic. The majority of immigrants were employed full-time (55.0%) and had lived in the U.S. for >10 years (73.7%). Around 46.7% of immigrants had a family income of more than USD 50,000, and 24.1% did not graduate from high school. Significant differences were found in demographic characteristics between e-cigarette ever and never users. About 71.8% of e-cigarette ever users were male, 38.2% had a bachelor’s degree, and 12.9% were at least 55 years old. Among e-cigarette never users, 48.3% were male, 32.3% had a bachelor’s degree, and 29.5% were at least 55 years old.

Table 2 presents the estimated prevalence of e-cigarette ever and current use by sociodemographic characteristics. Among immigrants, the prevalence of e-cigarette ever and current use increased significantly from 2.5% and 0.5% in 2014/2015 to 3.2% and 0.8% in 2018/2019, respectively. Among both male and female immigrants, the prevalence of e-cigarette use increased during the study period; however, the only significant increase was found for current e-cigarette use among female immigrants (76.8% change between 2014/2015 and 2018/2019). A significant increase in e-cigarette ever use was found for those aged 25–34 (59.0% change) and 35–44 years old (60.0% change), Hispanic immigrants (47.8% change), those employed full-time (43.3% change), and those who had a family income of more than USD 50,000 (41.1% change). Likewise, a significant increase was also found in current e-cigarette use prevalence for those who were younger than 35 years old, Hispanic immigrants, those employed full-time, and those with a family income of less than USD 25,000.

Among immigrants who had lived in the U.S. for between 6 and 10 years, the prevalence of e-cigarette ever use increased by 78.2% from 2.4% in 2014/2015 to 4.3% in 2018/2019. During the same period, the prevalence of current e-cigarette use increased by 284.7% (Table 2). Table 3 presents the association between years spent in the U.S. and e-cigarette ever and current use status. After adjusting for covariates, immigrants had statistically significantly lower odds of ever e-cigarette use compared to the mainland-born citizen (0–5 years in the U.S., Adjusted Odds Ratio (aOR) 0.57, 95% Confidence Interval (CI) 0.46–0.69; 6–10 years, aOR 0.51, 95% CI 0.41–0.63; 11–20 years, aOR 0.45, 95% CI 0.39–0.53; 20+years, aOR 0.68, 95% CI 0.62–0.76). Similar results were found for current e-cigarette use, with immigrants being less likely to be current users. In addition, those who used other tobacco products had significantly higher odds of being current (aOR 18.8, 95% CI 17.8–19.8) and ever (aOR 8.9, 95% CI 8.1–9.6) e-cigarette users. Additionally, lower odds of e-cigarette current and ever use were found among those aged less than 55 years old, who were male, Hispanic, and non-Hispanic Black, when compared to their reference categories. In both male and female immigrants (Table 4), similarly lower odds of e-cigarette current and ever use were found compared to mainland-born citizens, regardless of years spent in the U.S., although the odds ratios were generally smaller among females than males. 

## 4. Discussion

The current study investigated e-cigarette use among immigrants in the U.S. and whether the length of residence is associated with e-cigarette use when compared to U.S. natives. We found that the prevalence of current e-cigarette use among U.S. immigrants was significantly increased from 0.5% in 2014/2015 to 0.8% in 2018/2019, which is in contrast to a prior finding among the general population that current e-cigarette use was similar between 2014/2015 (2.3%) and 2018/2019 (2.4%) [24]. 

The prevalence of e-cigarette use was higher among males and young adults, which is in keeping with a previous study that found that male and young adults were more likely to use e-cigarette [25]. The gap in current e-cigarette use between male and female immigrants doubled from less than 0.4 percentage points in 2014/2015 to nearly 0.9 percentage points. Prior studies have indicated that males and young adults have lower odds of perceiving e-cigarettes as equally or more harmful than cigarettes [26,27,28,29]. The prevalence of e-cigarette use was highest among non-Hispanic white immigrants, consistent with findings in the general population. The 2018 National Health Interview Survey found that among U.S. adults, 16.9% of non-Hispanic white adults had ever used an e-cigarette, and 3.7% of them were current e-cigarettes users, while the prevalence was 10.0% and 1.6% among non-Hispanic Black adults, respectively [2]. 

Similar to findings from a recent study assessing the healthy migrant paradox (HMP) in cigarette smoking [20], we found that the prevalence of e-cigarette use in immigrant populations was significantly lower than that of the native-born population in the U.S., regardless of the length of residence. Elsewhere, a previous study found that naturalized U.S. citizens and non-U.S. citizens were less likely to be ever e-cigarette users than U.S. natives [11]. Another study among California adolescents found that non-citizens had significantly lower odds of lifetime e-cigarette use compared to U.S. citizens [30]. Our findings that e-cigarette use among all immigrants—regardless of years living in the U.S.—was consistently lower than among the native-born population run contrary to the notion that with increased length of stay, health behaviors between immigrants and native populations of the host country become similar [31]. In addition, the current study found that immigrants who had been in the U.S. for a shorter period had higher e-cigarette use than those who had been in the U.S. for more than 10 years. 

There are several possible explanations for low e-cigarette use prevalence among immigrants. It remains unclear to what extent existing home country regulatory approaches may have influenced immigrants’ smoking behavior before entering the U.S. Several countries have banned all types of e-cigarettes, and many others have more stringent sales laws [32]. Additionally, cultural buffering from the home country could be a protective factor against e-cigarette use [14,33]. Previous studies reported that Spanish speakers had lower e-cigarette use compared to English speakers [12,30,33]. Future qualitative studies could provide more insight into factors influencing immigrants’ smoking/vaping behaviors, and how marketing strategies—especially online marketing—might shape vaping behaviors. 

These findings are subject to some limitations. The self-reported nature of the survey may result in an inaccurate recall of events and experiences. Due to the study’s analytical design, causality claims cannot be made or implied. The TUS-CPS survey was conducted in two languages (English and Spanish), and it remains unclear how potential language barriers may have affected the survey results. It is also unclear whether immigrants who speak neither language were interviewed, leading to potential selection biases. This study also had many strengths; for example, we investigated a significant public health issue among a large sample of U.S. immigrants—a historically understudied population. Further, the large sample size allowed for an analysis of e-cigarette use by important sociodemographic characteristics. The current study did not study or discuss heterogeneity among immigrant subgroups. This study may not be generalizable to other countries, as the characteristics of the immigrants may be different. 

## 5. Conclusions

The current study examined e-cigarette use among immigrants in the U.S., and whether the length of residence is associated with e-cigarette use. Although we found a significant increase in e-cigarette use among U.S. immigrants from 2014/2015 to 2018/2019, e-cigarette use among all immigrants—regardless of years living in the U.S.—was consistently lower than among the native-born population. In contrast to the positive association between acculturation and cigarette smoking among some immigrant populations [17,19], our findings suggest that a longer length of stay in the host country was not associated with e-cigarette use. Further research may consider investigating whether the country of origin influences e-cigarette use during acculturation. 

## Figures and Tables

**Table 1:** Sample characteristics of all selected immigrants in the 2014/2015 and 2018/2019 Tobacco Use Supplement to the Current Population Survey (TUS-CPS)

Characteristics	Full Sample	E-Cigarette Ever User	E-Cigarette Never User
N	35,642	1005	34,208
Age			
18–24	6.9(6.5, 7.3)	14.6(11.3, 17.9)	6.6(6.2, 7.1)
25–34	21.0(20.5, 21.5)	35.0(31.4, 38.6)	20.6(20.1, 21.1)
35–44	22.7(22.2, 23.2)	22.4(19.5, 25.3)	22.8(22.3, 23.3)
45–54	20.3(19.8, 20.8)	15.1(12.7, 17.5)	20.5(20.0, 21.0)
55+	29.1(28.6, 29.6)	12.9(10.7, 15.1)	29.5(28.9, 30.0)
Sex			
Male	49.2(48.6, 49.8)	71.8(68.7, 75.0)	48.3(47.7, 49.0)
Female	50.8(50.2, 51.4)	28.2(25.0, 31.3)	51.7(51.0, 52.3)
Race			
Non-Hispanic white	17.4(17.0, 17.8)	33.4(30.1, 36.7)	16.8(16.4, 17.3)
Non-Hispanic black	8.7(8.3, 9.0)	5.5(3.6, 7.3)	8.8(8.4, 9.1)
Hispanic	45.8(45.2, 46.4)	32.5(28.9, 36.1)	46.2(45.6, 46.9)
Non-Hispanic other	28.1(27.6, 28.7)	28.7(25.2, 32.2)	28.2(27.6, 28.7)
Employment status			
Full-time	55.0(54.4, 55.6)	64.0(60.3, 67.7)	54.8(54.2, 55.4)
Part-time	9.7(9.4, 10.1)	12.6(10.0, 15.3)	9.6(9.3, 10.0)
Unemployed	3.4(3.2, 3.6)	4.3(2.8, 5.8)	3.3(3.1, 3.6)
Not in labor force	31.9(31.4, 32.5)	19.1(16.0, 22.1)	32.2(31.7, 32.8)
Income			
USD <25,000	25.3(24.8, 25.8)	22.9(19.7, 26.1)	25.3(24.8, 25.9)
USD 25,000–50,000	28.0(27.5, 28.6)	25.2(22.0, 28.5)	28.1(27.5, 28.7)
USD >50,000	46.7(46.1, 47.3)	51.9(48.2, 55.6)	46.6(46.0, 47.2)
Educational attainment			
Some high school or less	24.1(23.6, 24.6)	11.0(8.6, 13.5)	24.4(23.9, 25.0)
High school graduate or GED	24.5(24.0, 25.1)	20.0(16.9, 23.1)	24.7(24.1, 25.2)
Some college or associate’s degree	18.9(18.4, 19.4)	30.8(27.3, 34.4)	18.6(18.1, 19.1)
At least a bachelor’s degree	32.4(31.8, 33.0)	38.2(34.6, 41.8)	32.3(31.7, 32.9)
Other tobacco product use			
Yes	13.7(13.3, 14.1)	79.0(76.0, 82.0)	11.6(11.2, 12.0)
No	86.3(85.9, 86.7)	21.0(18.0, 24.0)	88.4(88.0, 88.8)
Years in U.S.			
0–5	12.3(11.9, 12.7)	16.6(13.8, 19.4)	12.2(11.7, 12.6)
6–10	14.0(13.5, 14.4)	15.9(12.8, 19.0)	14.0(13.5, 14.4)
11–20	29.0(28.5, 29.6)	27.9(24.5, 31.2)	29.1(28.5, 29.7)
20+	44.7(44.1, 45.3)	39.6(36.1, 43.2)	44.8(44.2, 45.4)
Survey year			
2014–2015	48.0(47.4, 48.6)	41.4(37.9, 45.0)	48.2(47.6, 48.8)
2018–2019	52.0(51.4, 52.6)	58.6(55.0, 62.1)	51.8(51.2, 52.4)

All variables are presented as weighted percentages; Rao–Scott chi-square tests were used to compare characteristics between the two groups (*p* < 0.01). GED: General Educational Development.

**Table 2:** Estimated prevalence of e-cigarette ever and current use in immigrants since 2014

	E-Cigarette Ever Use			E-Cigarettes Current Use		
	2014–2015	2018–2019	Change%	2014–2015	2018–2019	Change%
Full Sample	2.52(488)	3.29(517)	30.43 *	0.49(106)	0.76(117)	56.99 *
Age						
18–24	5.51(49)	6.95(43)	26.16	0.41(6)	2.27(15)	447.14 *
25–34	3.73(153)	5.94(186)	59.09 *	0.67(27)	1.38(42)	107.34 *
35–44	2.20(113)	3.52(135)	60.02 *	0.46(25)	0.82(30)	76.61
45–54	2.21(91)	2.13(75)	−3.71	0.49(23)	0.40(17)	−18.77
55+	1.28(82)	1.32(78)	3.73	0.38(25)	0.23(13)	−39.77
Sex						
Male	3.66(325)	4.85(352)	32.61	0.68(71)	1.20(86)	76.75 *
Female	1.44(163)	1.78(165)	23.13	0.30(35)	0.34(31)	11.62
Race						
Non-Hispanic white	5.16(209)	6.07(194)	17.78	1.01(47)	1.32(43)	30.26
Non-Hispanic black	1.34(21)	2.25(30)	67.13	0.24(4)	0.58(8)	143.51
Hispanic	1.66(129)	2.46(150)	47.76 *	0.32(28)	0.61(30)	90.27 *
Non-Hispanic other	2.62(129)	3.29(143)	25.40	0.50(27)	0.74(36)	47.15
Employment status						
Full-time	2.75(298)	3.95(358)	43.34 *	0.61(73)	0.99(85)	63.40 *
Part-time	3.46(68)	4.14(53)	19.60	0.52(13)	0.67(11)	28.03
Unemployed	3.47(22)	4.05(18)	16.62	0.49(4)	0.74(3)	50.60
Not in labor force	1.73(100)	1.77(88)	1.87	0.28(16)	0.37(18)	35.00
Income						
USD <25,000	2.56(136)	2.76(95)	7.70	0.31(20)	0.70(23)	123.72 *
USD 25,000–50,000	2.33(124)	2.92(115)	25.23	0.55(30)	0.71(27)	28.86
USD >50,000	2.63(228)	3.71(307)	41.10 *	0.56(56)	0.81(67)	45.37
Educational attainment						
Some high school or less	1.39(60)	1.29(43)	−6.82	0.21(11)	0.38(10)	86.08
High school graduate or GED	1.94(91)	2.78(99)	43.24 *	0.52(24)	0.69(23)	31.93
Some college or associate‘s degree	4.64(154)	4.88(135)	5.12	0.95(35)	1.16(34)	22.37
At least a bachelor’s degree	2.67(171)	4.07(219)	52.27 *	0.42(32)	0.81(43)	91.87 *
Other tobacco product use						
Yes	15.08(410)	18.97(387)	25.83 *	2.70(86)	4.12(80)	52.64 *
No	0.46(78)	0.93(129)	102.82 *	0.12(20)	0.25(36)	104.55 *
Years in U.S.						
0–5	3.62(75)	4.21(79)	16.54	0.62(13)	1.03(16)	66.36
6–10	2.40(67)	4.27(78)	78.18 *	0.34(11)	1.31(19)	284.66 *
11–20	2.39(128)	3.20(139)	33.41	0.31(22)	0.72(36)	132.16 *
20+	2.35(218)	2.81(221)	19.35 *	0.62(60)	0.56(46)	−10.10

The prevalence of e-cigarette ever and current use was estimated using survey weights. The presented count n was unweighted; * Change% was statistically significant at *p* < 0.05.

**Table 3:** Association between years spent in the U.S. and e-cigarette ever and current use

	E-Cigarette Ever Use	E-Cigarettes Current Use
Years in U.S.		
Mainland-born	Ref	ref
0–5	**0.57(0.46, 0.69)**	**0.48(0.31, 0.72)**
6–10	**0.51(0.41, 0.63)**	**0.48(0.30, 0.78)**
11–20	**0.45(0.39, 0.53)**	**0.37(0.27, 0.51)**
20+	**0.68(0.62, 0.76)**	**0.62(0.50, 0.75)**
Age		
18–24	**0.70(0.65, 0.75)**	**0.70(0.63, 0.79)**
25–34	**0.48(0.44, 0.52)**	**0.55(0.49, 0.62)**
35–44	**0.34(0.31, 0.36)**	**0.43(0.38, 0.49)**
45–54	**0.18(0.17, 0.19)**	**0.24(0.21, 0.27)**
55+	ref	Ref
Sex		
Male	**0.72(0.69, 0.75)**	**0.84(0.79, 0.90)**
Female	ref	Ref
Race		
Non-Hispanic white	ref	Ref
Non-Hispanic black	**0.46(0.43, 0.50)**	**0.44(0.38, 0.51)**
Hispanic	**0.75(0.70, 0.81)**	**0.63(0.55, 0.72)**
Non-Hispanic other	1.07(0.98, 1.17)	0.97(0.83, 1.13)
Employment status		
Full-time	ref	Ref
Part-time	**1.10(1.03, 1.17)**	**1.15(1.03, 1.28)**
Unemployed	**1.27(1.16, 1.39)**	**1.19(1.02, 1.39)**
Not in labor force	0.93(0.88, 0.98)	0.95(0.87, 1.04)
Income		
USD <25,000	ref	Ref
USD 25,000–50,000	**0.92(0.87, 0.97)**	0.97(0.88, 1.05)
USD >50,000	**0.77(0.73, 0.81)**	**0.82(0.75, 0.90)**
Educational attainment		
Some high school or less	ref	Ref
High school graduate or GED	**1.10(1.02, 1.18)**	1.11(0.98, 1.25)
Some college or associate‘s degree	**1.17(1.08, 1.26)**	1.10(0.97, 1.24)
At least a bachelor’s degree	**0.59(0.55, 0.64)**	**0.50(0.43, 0.58)**
Other tobacco product use		
Yes	**18.76(17.81, 19.76)**	**8.85(8.13, 9.63)**
No	ref	Ref
Survey year		
2014–2015	ref	Ref
2018–2019	**1.22(1.18, 1.27)**	**1.14(1.07, 1.22)**

Significant odds ratios (*p* < 0.05) are presented in boldface.

**Table 4:** Association between years spent in the U.S. and e-cigarette use by sex

E-Cigarette Ever Use			
Years in U.S.	Baseline Model	Male	Female
Mainland-born	Ref	Ref	ref
0–5	**0.57(0.46, 0.69)**	**0.61(0.47, 0.79)**	**0.44(0.31, 0.62)**
6–10	**0.51(0.41, 0.63)**	**0.59(0.46, 0.76)**	**0.33(0.22, 0.49)**
11–20	**0.45(0.39, 0.53)**	**0.52(0.43, 0.63)**	**0.32(0.25, 0.41)**
20+	**0.68(0.62, 0.76)**	**0.79(0.69, 0.91)**	**0.55(0.47, 0.64)**
**E-cigarettes current use**			
Years in U.S.	Baseline Model	Male	Female
Mainland-born	Ref	Ref	Ref
0–5	**0.48(0.31, 0.72)**	**0.46(0.27, 0.78)**	**0.50(0.25, 0.98)**
6–10	**0.48(0.30, 0.78)**	0.72(0.44, 1.17)	*
11–20	**0.37(0.27, 0.51)**	**0.43(0.29, 0.63)**	**0.26(0.16, 0.42)**
20+	**0.62(0.50, 0.75)**	**0.70(0.54, 0.91)**	**0.51(0.38, 0.68)**

Significant odds ratios (*p* < 0.05) were presented in boldface. * Small cell size.

## Data Availability

Publicly available.
